# Liposomal Iron vs. Conventional Iron in the Treatment of Iron Deficiency Anemia in Children: A Randomized Controlled Trial

**DOI:** 10.7759/cureus.97183

**Published:** 2025-11-18

**Authors:** Suresh Rangaraj, Preethi Suresh, Subash Sundar, Prasanna Raju

**Affiliations:** 1 Department of Pediatrics, SRM Medical College Hospital and Research Centre, SRM Institute of Science and Technology, Chengalpattu, IND

**Keywords:** conventional iron, elemental iron, ferrous ascorbate, iron deficiency anemia, liposomal iron

## Abstract

Introduction: Despite their established efficacy, gastrointestinal adverse effects of ferrous iron lead to reduced acceptability, poor adherence to treatment, and the persistence of iron deficiency anemia (IDA). Liposomal iron is characterized by high bioavailability, reduced side effects, and improved compliance, making it appropriate for patients requiring iron supplementation. Hence, there is a need to compare conventional iron (ferrous ascorbate) with liposomal iron employed for the treatment of IDA in children.

Aims and objectives: The study aimed to compare the effectiveness and tolerability of liposomal iron with conventional elemental iron (ferrous ascorbate) in improving hemoglobin levels and iron indices among children aged 6-60 months with IDA.

Methodology: The study was conducted between June and September 2024 at SRM Medical College Hospital and Research Centre in Tamil Nadu, India. Children aged 6-60 months with confirmed IDA, not receiving any iron supplementation for three months prior to study inclusion, and with no other hematological disorders or active infections were included. A total of 98 children were included and allocated into two groups of 49 each. Group A was treated with liposomal iron (1 mg/kg/day), and Group B with ferrous ascorbate (3 mg/kg/day) for one month. The response to treatment in both groups was assessed. Statistical analysis was done using the chi-square test and the t-test.

Results: Baseline characteristics were similar. The mean base hemoglobin in Group A was 8.4 g/dL, and that in Group B was 8.3 g/dL. After one month of iron supplementation, the mean hemoglobin only rose to 8.5 g/dL in Group A but to 9.3 g/dL in Group B. The difference in the rise of hemoglobin between Group A and Group B was significant (p=0.0018). Liposomal iron had adequate oral tolerance (p=0.042), better adherence (p=0.047), and a lesser incidence of constipation (p=0.046).

Conclusion: Despite poor oral tolerability, poor adherence, and a higher incidence of constipation, ferrous ascorbate was found to be superior compared to liposomal iron in treating children with IDA.

## Introduction

The Comprehensive National Nutrition Survey 2016-18 indicates that over 50% of childhood anemia is attributable to a dietary deficit [1]. The advised therapeutic dosage of conventional elemental iron (ferrous ascorbate) for children is 3-6 mg/kg/day [2,3]. It has been shown that dosages of 2-3 mg/kg/day of elemental iron are effective and enhance patient compliance by minimizing adverse effects, including stomach discomfort and constipation [4]. Despite their established efficacy, gastrointestinal adverse effects pose significant concerns with oral ferrous salts, impacting up to 32% of patients [5]. These complications can lead to reduced acceptability, poor adherence to treatment, and the persistence of iron deficiency anemia (IDA) [6].

Liposomal iron (ferric diphosphate) is a special delivery system where inorganic iron is encapsulated within tiny liposomes, enhancing the absorption of iron [7,8]. It is characterized by high bioavailability, reduced side effects, and improved compliance, making it appropriate for patients requiring iron supplementation who are intolerant to oral treatments [9]. Hence, there is a need to compare conventional iron (ferrous ascorbate) with liposomal iron employed for the treatment of IDA in children.

## Materials and methods

The study was conducted after obtaining permission from the Institutional Ethics Committee and after registering with the Clinical Trials Registry of India (CTRI/2024/06/068445) between June and September 2024 at SRM Medical College Hospital and Research Centre, Tamil Nadu, India.

Data source and study population

Children aged 6-60 months with confirmed IDA (as evidenced by hematological parameters: hemoglobin, serum ferritin, transferrin saturation, and serum iron), not receiving any iron supplementation for three months prior to study inclusion, and with no other hematological disorders or active infections were included. Children over 60 months of age, those with chronic conditions that include celiac disease, malabsorption syndromes, autoimmune gastritis, and other serious medical conditions, and individuals requiring blood transfusions were excluded from the study.

Based on a previous study, assuming a large effect size (d=0.8) with a significance level of 0.05 and a study power of 80%, the analysis using G*Power (Heinrich-Heine-Universität Düsseldorf, Düsseldorf, Germany) determined that a minimum of 52 participants (26 per group) were necessary to achieve statistical significance [10]. To account for potential attrition, the final sample size was increased to 49 per group. In our study, a total of 98 children were included and allocated into two groups - Group A and Group B - of 49 each, using computer-generated random numbers in a 1:1 ratio. Group A children were treated with oral liposomal iron suspension (ferric diphosphate formulated using micronized iron, containing 10 mg of iron in 5 mL) licensed and available commercially, prescribed at a dose of 1 mg/kg/day (one-third of the conventional iron dose was given based on previous recommendation) [11]. Group B children received ferrous ascorbate suspension at a dose of 3 mg/kg/day. The study was conducted without blinding. The response to treatment in both groups was assessed by measuring serum iron, serum ferritin, transferrin saturation, and hemoglobin after 30 days of treatment. Additionally, the two groups were compared in terms of the incidence of oral tolerability, constipation, and diarrhea. Adherence was assessed through parental self-report and categorized as good if fewer than or equal to two doses/week were missed and as bad if more than two doses/week were missed.

Data collection and study variables

A comprehensive history encompassing antenatal iron supplementation, gestational age at delivery, birth weight, exclusive breastfeeding, age at the commencement of complementary feeding, improper techniques of complementary feeding, and daily intake of cow's milk was collected from the mother or caregiver. Anthropometric parameters and iron indices, including serum iron, serum ferritin, transferrin saturation, and hemoglobin, were evaluated before the initiation of the trial and one month after treatment. The adherence and adverse effects, such as diarrhea, constipation, and oral tolerability, were compared between the two groups.

Ethical considerations

Institutional Ethical Committee approval was obtained before the start of the study (approval number: SRMIEC-ST0723-953). Informed written consent was obtained from each participant. Additionally, the study was registered in the Clinical Trials Registry of India (CTRI/2024/06/068445).

Data handling and statistical analysis

Statistical analysis was performed using IBM SPSS Statistics Version 26.0 (IBM Corp., Armonk, USA). The continuous variables were expressed as mean and standard deviation (SD). Categorical variables were expressed as frequency and percentage. A chi-square test was used to analyze the categorical variables. An independent t-test was used for continuous variables to demonstrate the significant differences between groups. A p-value <0.05 was considered statistically significant.

## Results

**Figure 1:**
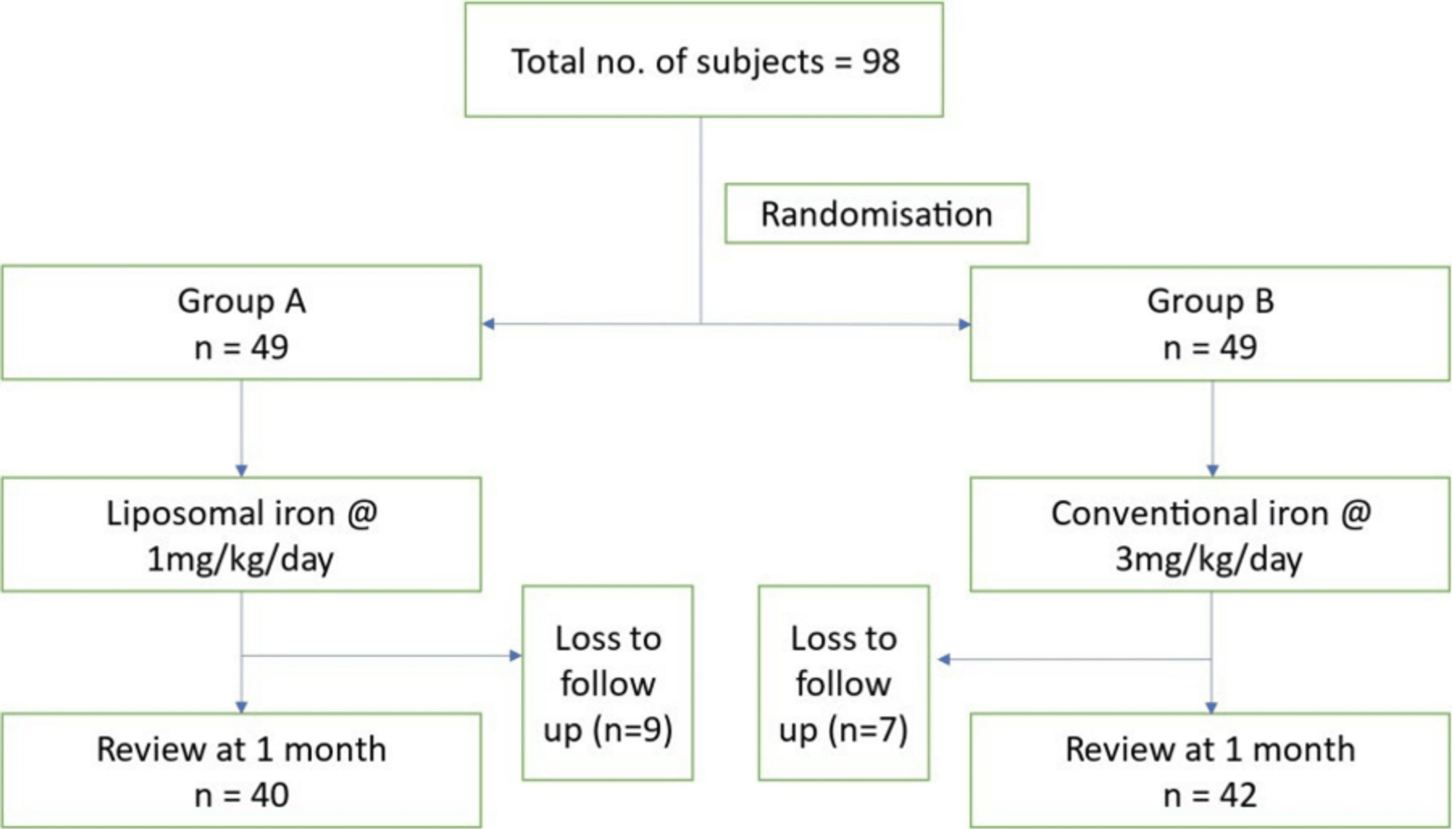
Schematic representation of the study flow

A total of 16 children were lost to follow-up, nine in Group A and seven in Group B. After one month of treatment, 40 children in Group A and 42 children in Group B were analyzed, as shown in Figure 1.

The baseline characteristics of the research participants were similar. No significant difference in the incidence of IDA was noted between male and female participants. Children with a history of early complementary feeding introductions and improper complementary feeding techniques had a higher frequency of IDA. Nonetheless, other evaluated risk factors for IDA, such as dietary habits, exclusive breastfeeding, complementary feeding, daily cow's milk intake, antenatal iron supplementation, and gestational age at delivery, had no significant differences between groups, as shown in Table 1.

**Table 1:** Baseline characteristics of the study subjects (n=98) Age is presented as mean ± standard deviation (SD), while other values are presented as N (%). Chi-square test and independent t-test were used. A p-value <0.05 was considered statistically significant.

Baseline Characteristics		Group A - Liposomal Iron (n=49)	Group B - Ferrous Ascorbate (n=49)	t-value / ꭓ^2^ value	p-value
Age in years		2.16 ± 1.38	1.79 ± 0.92	1.59	0.117
Sex	Male	28 (57.1%)	22 (44.9%)	1.47	0.225
	Female	21 (42.9%)	27 (55.1%)		
Diet	Veg	11 (22.4%)	13 (26.5%)	0.22	0.638
	Non-veg	38 (77.6%)	36 (73.5%)		
Exclusive breastfeeding	Yes	26 (53.1%)	25 (51.0%)	0.04	0.84
	No	23 (46.9%)	24 (49.0%)		
Early complementary feeding	Yes	27 (55.1%)	30 (61.2%)	0.38	0.539
	No	22 (44.9%)	19 (38.8%)		
Inappropriate complementary feeding	Yes	25 (51.0%)	27 (55.1%)	0.17	0.686
	No	24 (49.0%)	22 (44.9%)		
Daily intake of cow’s milk	<200 mL	12 (24.5%)	15 (30.6%)	0.29	0.865
	200-400 mL	25 (51.0%)	23 (46.9%)		
	>400 mL	12 (24.5%)	11 (22.4%)		
Antenatal iron supplementation	Yes	40 (81.6%)	37 (75.5%)	0.54	0.460
	No	9 (18.4%)	12 (24.5%)		
Gestation	Term	40 (81.6%)	39 (79.6%)	0.07	0.798
	Preterm	9 (18.4%)	10 (20.4%)		

The baseline anthropometry of both groups was comparable. The serum iron, serum ferritin levels, transferrin saturation, and mean hemoglobin in Group A were 12.0 ± 9.3 μg/dL, 11.4 ± 8.1 μg/L, 9.6 ± 1.2, and 8.49 ± 1.028 g/dL, respectively, and in Group B, they were 13.2 ± 6.1 μg/dL, 10.5 ± 7.3 μg/L, 9.2 ± 2.6, and 8.33 ± 1.04 g/dL. The p-values of 0.494, 0.599, 0.371, and 0.486, respectively, indicate the absence of a significant difference in the baseline serum iron, serum ferritin levels, transferrin saturation, and hemoglobin between the two groups, as shown in Table 2.

**Table 2:** Baseline anthropometry and iron indices (n=98) Values are presented as mean ± standard deviation (SD). Independent t-test was used. A p-value <0.05 was considered statistically significant.

Anthropometry and Iron Indices	Group A - Liposomal Iron (n=49)	Group B - Ferrous Ascorbate (n=49)	t-value	p-value
Height	84.96 ± 17.05 cm	84.22 ± 11.10 cm	0.27	0.791
Weight	12.80 ± 13.81 kg	10.46 ± 2.77 kg	1.75	0.085
Head circumference	46.45 ± 2.77 cm	45.29 ± 2.15 cm	1.57	0.121
Mid-arm circumference	12.27 ± 1.03 cm	12.26 ± 4.24 cm	1.00	0.319
Serum iron	12.0 ± 9.3 μg/dL	13.2 ± 6.1 μg/dL	0.69	0.494
Serum ferritin	11.4 ± 8.1 μg/L	10.5 ± 7.3 μg/L	0.53	0.599
Transferrin saturation	9.6 ± 1.2 %	9.2 ± 2.6 %	0.90	0.371
Hemoglobin	8.49 ± 1.028 g/dL	8.33 ± 1.04 g/dL	0.70	0.486

After one month of iron supplementation, the children in Group A did not exhibit a significant rise in iron indices, but the children in Group B exhibited a statistically significant elevation in iron indices as compared to Group A. This elevation in Group B indicates that the interventions or variables associated with this group are more efficacious in improving the iron indices, as shown in Table 3.

**Table 3:** Iron indices after one month of treatment Values are presented as mean ± standard deviation (SD). Independent t-test was used. A p-value <0.05 was considered statistically significant. * indicates statistical significance.

Iron Indices	Group A - Liposomal Iron (n=40)	Group B - Ferrous Ascorbate (n=42)	t-value	p-value
Serum iron	23.5 ± 6.3 μg/dL	37.4 ± 7.3 μg/dL	9.24	<0.0001*
Serum ferritin	21.7 ± 5.6 μg/L	40.9 ± 10.8 μg/L	10.17	<0.0001*
Transferrin saturation	17.0 ± 8.8 %	31.2 ± 9.4 %	7.06	<0.0001*
Hemoglobin	8.565 ± 1.072 g/dL	9.307 ± 1.003 g/dL	3.23	0.0018*

We also compared the adverse effect profile of conventional elemental iron against liposomal iron, and it was observed that liposomal iron exhibited superior oral tolerance (p=0.042), a lesser incidence of constipation (p=0.046), and better adherence (p=0.047) compared to ferrous ascorbate; however, no difference of statistical significance was observed in the incidence of diarrhea between the two groups (p=0.366), as shown in Table 4.

**Table 4:** Comparison of adherence and adverse effects Values are presented as N (%). Chi-square test was used. A p-value <0.05 was considered statistically significant. * indicates statistical significance.

Adherence and Adverse Effects		Group A - Liposomal Iron (n=40)	Group B - Conventional Iron (n=42)	ꭓ^2^ value	p-value
Diarrhea	Present	8 (20.0%)	12 (28.6%)	0.816	0.366
	Absent	32 (80.0%)	30 (71.4%)		
Constipation	Present	15 (37.5%)	25 (59.5%)	3.977	0.046*
	Absent	25 (62.5%)	17 (40.5%)		
Adherence	Good	32 (80%)	24 (57.1%)	4.943	0.047*
	Bad	8 (20%)	18 (42.9%)		
Oral tolerability	Good	27 (67.5%)	19 (45.2%)	4.123	0.042*
	Bad	13 (32.5%)	23 (54.8%)		

## Discussion

In our study, liposomal iron supplementation did not increase hemoglobin and other iron indices significantly in children with IDA, whereas ferrous ascorbate supplementation significantly improved the iron indices in these children. Nevertheless, the evaluation indicated superior oral tolerability, a lesser occurrence of constipation, and better adherence of liposomal iron relative to traditional iron; however, no such difference was noted in the incidence of diarrhea.

The study results were similar to those of Russo et al. (2020), who aimed to monitor oral iron treatment in children aged between three months and 12 years with IDA. The hemoglobin improvement between two and eight weeks demonstrated a greater median increase in the ferrous groups relative to the others. The highest occurrence of gastrointestinal side effects was noted with ferrous salts, while liposomal iron and ferric iron exhibited no adverse effects [12].

Research analogous to ours was executed by Kiliç et al. (2024), who intended to evaluate the effectiveness of several oral iron formulations given for the prevention of IDA in healthy infants. This retrospective analysis included babies aged 6-12 months; iron prophylaxis (ferric, ferrous, or liposomal iron) was given at four months of age, and complete blood count and serum ferritin level evaluations were done in a 6-12-month period. The mean hemoglobin levels were markedly elevated in the ferrous group as compared to the ferric and liposomal iron groups (p=0.008) [13].

The results of our study conflicted with those of Kulkarni and Menon (2023), who examined blood iron concentrations in healthy adult female participants on Tasiron tablets containing 30 mg of micronized liposomal ferric diphosphate, compared to those receiving tablets with 100 mg (elemental iron) of ferrous ascorbate, for a duration of 15 days. The liposomal group was provided with one-third of the iron dosage given to the control group. The study demonstrated that liposomal iron at a dosage of one-third (30 mg) was similarly efficient as conventional elemental iron supplementation (100 mg) in enhancing iron levels by day 16 (p<0.05). The elevation in hemoglobin levels on day eight was greater in the group administered liposomal iron than in the group receiving ferrous ascorbate [14].

Furthermore, our results were in contrast to those of Parisi et al. (2017), who investigated various iron supplementation regimens on maternal hematological state and pregnancy outcomes in 80 healthy pregnant women. The studied formulations consisted of ferrous formulations and liposomal iron administered at two distinct dosages, compared to a control group. Both liposomal groups had markedly elevated hemoglobin and ferritin levels in comparison to the control group, with a p-value of <0.01. The study indicated that liposomal iron had benefits comparable to greater doses of ferrous iron on maternal hematological parameters [15].

Our study findings also conflicted with those of Indriolo and Ravelli (2014), who conducted research to examine the efficiency and safety of liposomal iron and ferrous sulfate in patients with IDA and inflammatory bowel disease. The study indicated that a rise in hemoglobin >2 g/dL was more common in patients treated with liposomal iron than in controls and was equivalent to patients treated with ferrous sulfate [11]. Comparisons of liposomal iron and conventional elemental iron in various studies are depicted in Table 5.

**Table 5:** Comparison of liposomal iron and conventional iron in various studies IBD: irritable bowel syndrome; IDA: iron deficiency anemia

Study and Year	Study Population	n	Aim	Results
Indriolo and Ravelli (2014) [11]	IBD patients with IDA	17	Efficiency of liposomal and ferrous sulfate in IBD patients with IDA	Liposomal iron = ferrous iron
Russo et al. (2020) [12]	Children: 3 months - 12 years	107	Monitor oral iron treatment	Ferrous salts > liposomal iron
Kiliç et al. (2024) [13]	Infants: 6 months - 12 months	371	Effectiveness of oral iron formulations for IDA prophylaxis	Ferrous salts > liposomal iron (p=0.008)
Kulkarni and Menon (2023) [14]	Healthy adult women	14	Liposomal iron vs. ferrous iron	Liposomal iron = ferrous iron (p<0.05)
Parisi et al. (2017) [15]	Healthy pregnant women	80	Liposomal iron vs. ferrous iron	Liposomal iron = ferrous iron (p<0.01)
Our study	Infants: 6 months - 60 months	98	Effectiveness of liposomal iron vs. ferrous iron in IDA	Ferrous ascorbate > liposomal iron in treating children with IDA

In contrast to articles endorsing the advantages of liposomal iron compared to conventional iron (ferrous ascorbate), our research offers no compelling evidence to support this claim. Our study unequivocally ascertains the advantages of ferrous iron in treating IDA in children despite its poor tolerability and higher incidence of constipation.

Limitations of the study

The modest sample size limits the statistical power of the study, and the short duration of follow-up restricts the ability to evaluate the long-term efficacy, safety, and sustainability of the observed effects. The study was not blinded, which could have introduced participant or observer bias, especially for subjective parameters such as adherence and tolerability. Additionally, the study did not provide detailed descriptions of the laboratory testing methods used for hematological and biochemical parameters, which may limit exact reproducibility. 

## Conclusions

Despite poor oral tolerability, poor adherence, and a higher incidence of constipation, ferrous ascorbate was found to be superior compared to liposomal iron in treating children with IDA. However, further research with varied demographics and contexts, large sample sizes, and extended follow-ups is essential to assess the efficacy of oral liposomal iron.
